# The nature of functional variability in plantar pressure during a range of controlled walking speeds

**DOI:** 10.1098/rsos.160369

**Published:** 2016-08-17

**Authors:** Juliet McClymont, Todd C. Pataky, Robin H. Crompton, Russell Savage, Karl T. Bates

**Affiliations:** 1 Institute of Ageing and Chronic Disease, William Duncan Building, L7 8TX Liverpool, UK; 2 Institute for Fiber Engineering, Shinshu University, Ueda, Japan

**Keywords:** functional variability, plantar pressure, speed, biomechanics, step-to-step variation, pedobaragraphic statistical parametric mapping

## Abstract

During walking, variability in step parameters allows the body to adapt to changes in substrate or unexpected perturbations that may occur as the feet interface with the environment. Despite a rich literature describing biomechanical variability in step parameters, there are as yet no studies that consider variability at the body–environment interface. Here, we used pedobarographic statistical parametric mapping (pSPM) and two standard measures of variability, mean square error (m.s.e.) and the coefficient of variation (CV), to assess the magnitude and spatial variability in plantar pressure across a range of controlled walking speeds. Results by reduced major axis, and pSPM regression, revealed no consistent linear relationship between m.s.e. and speed or m.s.e. and Froude number. A positive linear relationship, however, was found between CV and walking speed and CV and Froude number. The spatial distribution of variability was highly disparate when assessed by m.s.e. and CV: relatively high variability was consistently confined to the medial and lateral forefoot when measured by m.s.e., while the forefoot and heel show high variability when measured by CV. In absolute terms, variability by CV was universally low (less than 2.5%). From these results, we determined that variability as assessed by m.s.e. is independent of speed, but dependent on speed when assessed by CV.

## 1. Introduction

Walking is a complex task involving the coordination of multiple body segments over multiple cycles (steps) [1–3]. As one of the most practised of all motor skills [4], variability allows us to continuously construct adaptive coordination patterns as we move through the environment, resisting expected and unexpected perturbations that may occur causing instability [5,6]. Studies of variability are thus central to understanding gait stability [7–9]. Control of speed during walking is a coordinated task driven by the interactions of the nervous and musculoskeletal systems and the environment [10]. Motor tasks such as a step cycle are provided a window of optimal variability that enables accurate, stable completion of that task [11], despite internal and external perturbations.

Variability in kinematic parameters is not always considered beneficial, but equally some degree of variability is not always considered detrimental [9,12]. High variability in kinetic and kinematic variables can increase energetic costs [13], and increase the risk of falls [14], but equally it can facilitate an increase in motor performance [15]. Both younger (less than 65 years) and older (more than 65 years) adults show increases in the variability in step parameters when walking at speeds faster, or slower than their comfortable walking speed [4]; only there is a slightly wider distribution in the magnitude of variability in older adults [12]. Thus, the nature of the relationship between motor control and biomechanical variability is not simple, and is not yet completely understood; for example, we do not understand how variability functions at the body–ground interface. Fortunately, plantar pressure records capture the summation of kinetic and kinematic forces produced by the moving body against the ground. Fluctuations in the centre of mass over the base of support, and perturbations that challenge stability and balance during adaptive coordination, occur here at the foot–ground interface. It is therefore timely for a systematic study characterizing variability in plantar pressure, and this is the goal of this contribution.

The variable nature of motor patterns and the potential for a change in substrate or speed with each step imply that each pressure record will be slightly different [16,17]. A recent study of plantar pressure at a single walking speed using approximately 500 records per subject, demonstrated large inter- and intra-subject (i.e. step-to-step) pressure variation in the midfoot [18]. However, discussion of variability across the whole plantar surface and the influence of speed were not considered as part of that contribution.

Here therefore, we compare the magnitude and spatial variability in plantar pressure records across a wide range of controlled walking speeds (1.1–1.9 m s^−1^) using a large dataset. Most previous investigations of the effects of speed on peak plantar pressure distribution use self-selected walking speeds, described as ‘slow’, ‘preferred’ and ‘fast’ [19–30]. Here, we control for speed in order to standardize the comparative analysis between walking speeds. We applied two calculators of variability to this dataset: mean square error (m.s.e.) and coefficient of variation (CV) at a pixel level across the whole plantar surface, using pedobarographic statistical parametric mapping (pSPM). The aims of this study were to assess the effect of walking speed (1.1–1.9 m s^−1^) on the: (i) magnitude and (ii) spatial distribution of variability across the whole plantar surface of the foot and (iii) to describe the spatial distribution of variability using these two commonly used metrics in biomechanical studies, m.s.e. and CV.

## 2. Material and methods

A total of 16 subjects (11 male, 5 female, aged 21–47 years; table 1), without pathologies, abnormalities or injuries, walked barefoot on a Zebris FDM-THM plantar pressure sensing treadmill at controlled speeds of 1.1 m s^−1^, 1.3 m s^−1^, 1.5 m s^−1^, 1.7 m s^−1^ and 1.9 m s^−1^ for 5 min in a randomized trial order. The slowest speed (1.1 m s^−1^) was selected as slower than a ‘comfortable’ walking speed for healthy, young cohorts. We are conscious that comfortable speed is relative to different ages and abilities; however, 1.1 m s^−1^ may generally be considered slow for healthy cohorts. The fastest speed, 1.9 m s^−1^, was chosen as the closest speed to the accepted walk/run transition (defined as 1.88 m s^−1^ [31]). 1.3 m s^−1^, 1.5 m s^−1^ and 1.7 m s^−1^ were chosen for intuitive ease of analysis.

**Table 1.** Summary of subjects' anthropomorphic measurements and *N* pressure records collected at each walking speed. Leg length was measured as the distance from the greater trochanter to the plantar foot surface.

			weight	height	leg length	*N*	*N*	*N*	*N*	*N*	total
subject	gender	age	(kg)	(m)	(m)	(1.1 m s^−1^)	(1.3 m s^−1^)	(1.5 m s^−1^)	(1.7 m s^−1^)	(1.9 m s^−1^)	*N*
A	M	21	75	1.74	0.905	528	583	626	668	694	3099
B	M	28	78.6	1.91	1	526	544	577	613	622	2882
C	F	24	59	1.65	0.83	574	600	628	687	721	3210
D	M	27	83.3	1.85	0.925	516	558	593	641	688	2996
E	M	22	82.7	1.96	0.965	475	533	562	608	646	2824
F	M	26	86	1.75	1.3	486	520	562	590	622	2780
G	M	29	70	1.75	0.885	533	591	607	644	702	3077
H	M	30	87	1.7	0.905	526	568	568	654	665	2981
I	F	31	52.7	1.58	0.85	560	612	651	694	746	3263
J	M	31	74	1.73	0.92	502	553	586	651	707	2999
K	M	44	80	1.93	1	504	573	597	630	660	2964
L	F	37	63	1.63	0.94	529	577	594	622	649	2971
M	F	37	52.7	1.56	0.83	611	663	692	751	818	3535
N	M	24	81	1.75	0.89	523	563	614	646	686	3032
O	F	23	64.4	1.58	0.85	578	614	687	708	765	3352
P	M	20	73	1.85	0.96	513	571	599	624	659	2966
										total *N*	48 931

Peak pressure values from each sensor contacted on the treadmill, within the boundaries of each foot, were extracted using a custom-written C program [28,29], yielding between 2780 and 3535 peak pressure images (p-images) per subject across the five speed trials (table 1). This exceeds the required sample size of 400 steps suggested for reliability in studies of variability in lower extremity kinematic parameters [31]. All p-images within each speed trial were registered to each other in a vertical stack using a two-stage rigid body transformation via an algorithm that minimized the m.s.e. between the images, such that homologous structures optimally overlapped [32]. The first step recorded during each subject's walking trials was used as the registration template to which all subsequent images in each speed trial were registered. The mean image from each speed trial was calculated from the stack and used as the registration template for a second iteration of the same dataset [28].

Intra-subject analyses using pSPM were conducted on all pressure records to quantify spatial variability at the five controlled walking speeds [32]. To quantitatively compare variability in peak pressure at different speeds, we used two different measures of variability. The algorithm used to register p-images in pSPM is based on minimizing the m.s.e. between pixels globally, across each image. Previous studies of walking speed using pSPM have sought to test if the mean pressure across the foot differs across walking speeds [22,28]. Thus, in the context of previous studies using pSPM that examine changes in pressure with speed [28] it is logical to assess variability relative to the mean pressure in each pixel at each speed. The m.s.e. reflects the absolute variation of each pixel value from the same pixel value in the mean p-image. In addition, we chose to calculate the CV as a widely used metric to quantify variability in clinical [1,7,33–35] and sports biomechanics [36–40]. CV calculates the variance of the entire sample about the mean but cannot take into consideration the error that arises from differences in pixel vector values. However, the widespread use of CV to quantify variability in kinematic parameters during gait allows us to compare levels of variability in plantar pressure to other biomechanical parameters (e.g. step length, width, time and impulse).

The m.s.e. was calculated over non-zero pixels in each p-image within a subject's total sample according to

$$\text{m.s.e}.=\frac{1}{N}\sum {\left(I_{0}k-I_{k}k\right)}^{2},$$

where *N* is the total number of non-zero pixels in the mean image, *I*_0_ is the mean of the subject's overall sample and *I_k_* is an individual pedobarographic record. The CV was calculated over non-zero pixels in each pedobarographic image within a subject's total sample according to

$$\mathrm{CV}=\frac{m}{s.d.}\times 100,$$

where *m* is the mean value of each non-zero pixel over all pressure images in the sample, and s.d. is the standard deviation of the sample across all p-images in each subject's dataset. Each value is multiplied by 100 to produce a percentage classed as high (more than 5%) or low (less than 5%) [41]. Very low levels of variability are considered less than 3% [4].

The m.s.e. and CV of each non-zero pixel is summed to produce a total m.s.e. and CV value for each individual pressure record, about the subject's overall mean pressure image. Each speed trial produces a different number of prints as more steps are generally taken at faster speeds. To standardize our comparisons we used a downsampling approach to extract random sub-samples of 400 prints from the overall *n*, a total of 100 times for each speed and calculated the mean m.s.e. and CV of these 100 samples of 400 prints. This value was retained as representative for each speed. To assess changes in variability with speed, we plotted m.s.e. and CV against speed using reduced major axis (RMA) regression, and tested for linear changes with speed. In addition, we conducted topological regression analysis on the m.s.e. analysis, to test for linear changes in variability across the plantar surface with speed. All image processing and analysis described above was conducted using Matlab (MathWorks, USA).

## 3. Results

**Figure 1.**
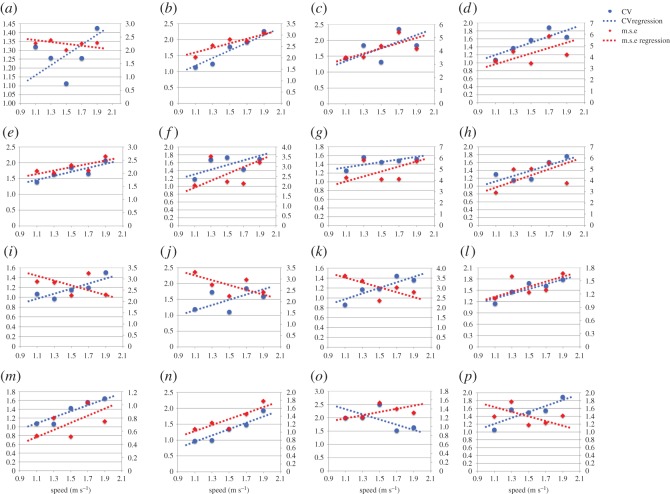
(*a*–*p*) RMA regression suggested positive linear trends in m.s.e. (*y axis*) across all walking speeds in 11 out of 16 subjects, and CV (*z axis*) and speed in 15 out of 16 subjects. However *r*^2^ and *p*-values only strongly support a linear increase in plantar pressure variability with speed in four subjects when assessed by m.s.e., but in 10 subjects when assessed by CV (*r*^2^ > 0.75, *p* = <0.05) (see table 2).

**Figure 2.**
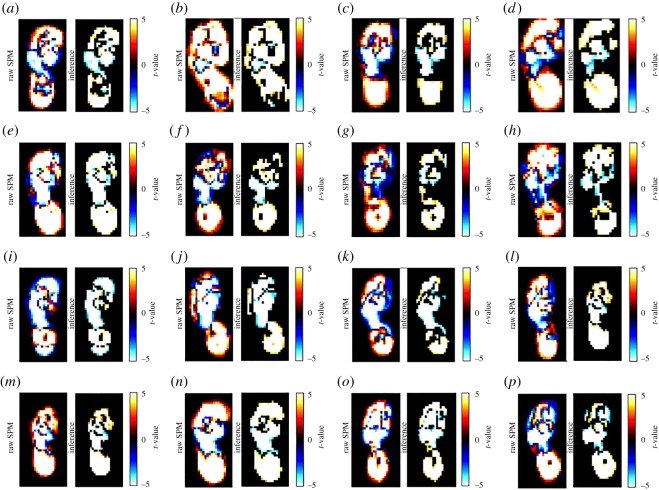
(*a*–*p*) Linear regression by pSPM (left panel) reveals no statistical support for linear changes in variability by m.s.e. with speed. The left image is the inference map, showing areas of increasing or decreasing variability in pressure across the plantar surface. The red pixels indicate that variability is increasing with speed around the periphery of the heel and forefoot only. The blue pixels indicate that variability is decreasing with speed around periphery of the midfoot and occasionally the hallux. Statistically significant pixels are concentrated almost exclusively around the periphery of mechanically distinct areas of the foot, thus they are most probably artefacts of small changes in foot contact area, size and shape with speed. The mechanically distinct areas of the plantar surface, heel, mid- and forefoot, remain white with no statistically significant relationships evident between variability and speed.

**Figure 3.**
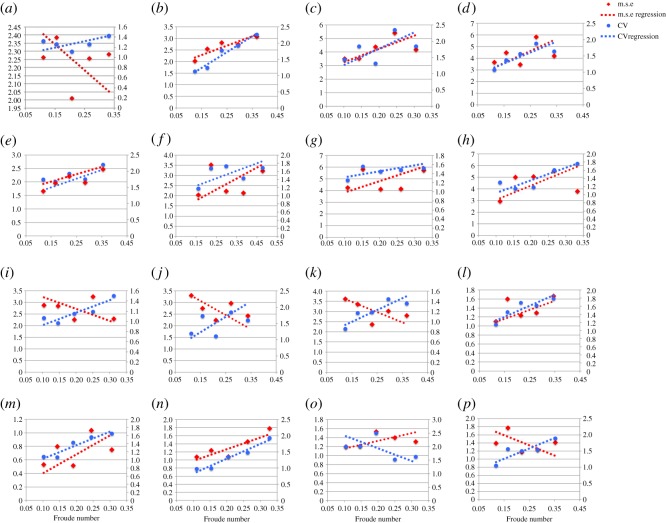
(*a*–*p*) RMA regression suggested positive linear relationships between m.s.e. (*y axis*) and Froude number in 11 out of 16 subjects, and CV (*z axis*) and Froude number in 15 out of 16 subjects. However, *R*^2^ and *p*-values (*r*^2^ > 0.75, *p* = <0.05) strongly support linear increases in variability with Froude in only four subjects when assessed by m.s.e. in four subjects, and in 11 subjects when assessed by CV (table 3). Froude number is (Fr = *v*^2^/*g* × LL), where *v*^2^ is speed squared, *g* is gravity (9.81 m s^−2^) and LL is each subject's leg length measured from the superior apex of the iliac crest to the where the heel meets the floor.

**Figure 4.**
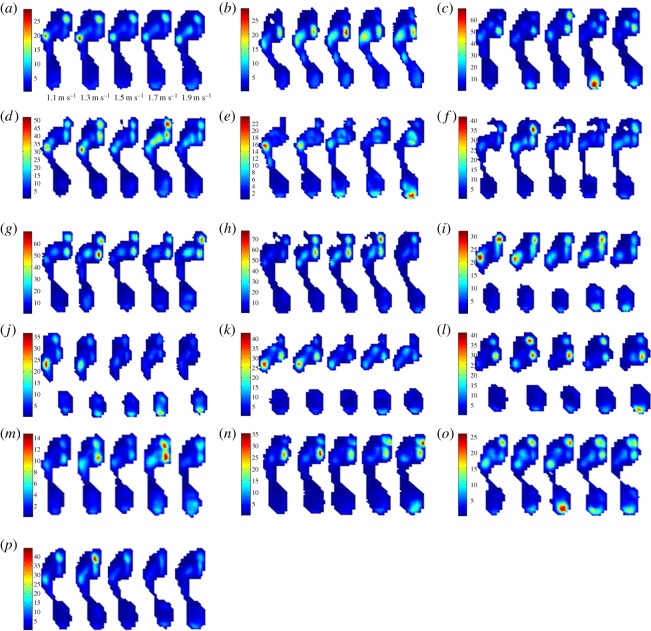
(*a*–*p*) m.s.e. variation maps represent the distribution and magnitude of the combined mean m.s.e. in each pixel across the plantar surface of the foot at each speed trial in all subjects. Intra-subject spatial variability measured by the mean m.s.e. is highest and confined almost exclusively to the lateral and medial forefoot in all subjects; however, there is no consistent increasing or decreasing relationship between mean m.s.e. and speed.

**Figure 5.**
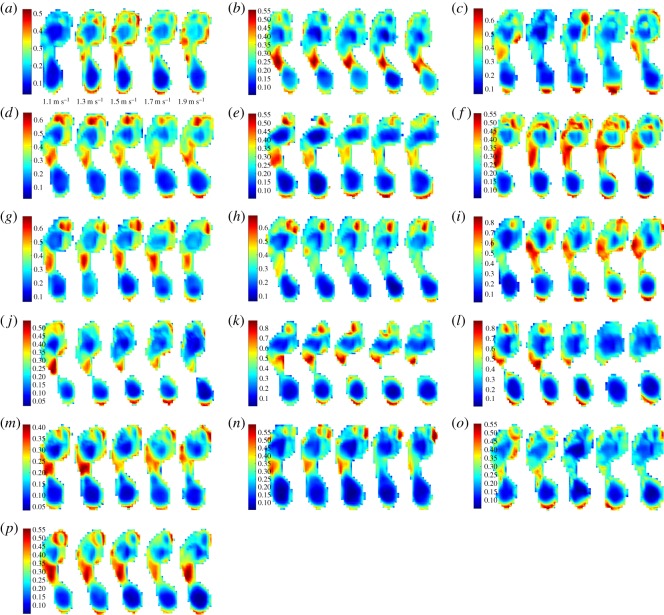
(*a*–*p*) CV variation maps represent the distribution and magnitude of the combined CV in each pixel across the plantar surface of the foot at each speed trial in all subjects. Intra-subject spatial variability measured by the CV is highest under the big toe and medial phalanges, as well as in the midfoot. There is no consistent increasing or decreasing relationship between mean CV and speed.

The main results of this study can be summarized as follows:

(i) The magnitude of variability in plantar pressure assessed by m.s.e. is disparate and observed between speeds, and between subjects (figure 1). All subjects expressed CV less than 2.5% (figure 1), which is less than that reported as ‘very low’ by standard measures of CV in biomechanics literature (less than 3%).
(ii) RMA regression suggested positive linear trends in m.s.e. across walking speeds in 11 out of 16 subjects, and CV and speed in 15 out of 16 subjects (figure 1). *R*^2^ and *p*-values strongly support a linear increase in plantar pressure variability with speed when assessed by m.s.e. in 4 subjects, and in 11 subjects when assessed by CV (*R*^2^ > 0.5, *p* = <0.05) (table 2) (figure 1). Topological regression analysis of m.s.e. and speed confirmed no statistical support for a systematic linear increase in variability with speed (figure 2).
(iii) RMA regression suggested positive linear relationships between m.s.e. and Froude number in 11 out of 16 subjects, and CV and Froude number in 15 out of 16 subjects (figure 3). However, *R*^2^ and *p*-values (*R*^2^ > 0.5, *p* = <0.05) only strongly support linear increases in variability with Froude number when assessed by m.s.e. in 4 subjects, but in 11 subjects when assessed by CV (table 2) (figure 3).
(iv) Qualitative analysis of topological variation maps suggests that spatial distribution of m.s.e. is highest in the forefoot, specifically the lateral and medial margins, under metatarsal heads five and one, respectively, in all subjects, and is independent of speed (figure 4).
(v) Qualitative analysis of topological variation maps suggests that the spatial distribution of CV is highest in the midfoot, medial phalanges, big toe and lateral margins of the heel in all subjects, and is independent of speed (figure 5).

**Table 2.** Regression statistics reveal a linear increase in m.s.e. with speed in only four subjects (subjects B, E, L, N). When assessed by CV, regression statistics reveal a linear increase in CV with speed in 10 subjects (subjects B, D, E, H, I, K, L, M, N, P).

subject	calculation	*R*^2^	*p*-values	slope	intercept
A	m.s.e.	0.010181	0.87175	−0.43955	2.8989
	CV	0.084309	0.63556	0.35829	0.7358
B	m.s.e.	0.85392	0.024825	1.2425	0.77694
	CV	0.96216	0.024825	1.5003	−0.58675
C	m.s.e.	0.42412	0.23387	2.47	0.49312
	CV	0.26087	0.37919	1.2834	−0.17
D	m.s.e.	0.17387	0.48491	2.9502	−0.10196
	CV	0.73986	0.061446	0.9652	0.05617
E	m.s.e.	0.54797	0.15257	0.80283	1.0234
	CV	0.73153	0.064625	0.79805	0.51636
F	m.s.e.	0.049747	0.71839	2.1586	2.1586
	CV	0.27677	0.36249	0.73674	0.43294
G	m.s.e.	0.051614	0.71324	2.7744	0.63995
	CV	0.36376	0.28157	0.37231	0.89058
H	m.s.e.	0.10784	0.58952	3.4058	−0.64398
	CV	0.64391	0.10223	0.86014	0.10809
I	m.s.e.	0.081786	0.081786	−1.3333	4.711
	CV	0.73681	0.062601	0.64107	0.21239
J	m.s.e.	0.33237	0.30894	−1.3371	4.742
	CV	0.20008	0.45008	1.0392	−0.068518
K	m.s.e.	0.4088	0.24542	−1.5495	5.3533
	CV	0.80221	0.039817	0.71056	0.13506
L	m.s.e.	5.84 × 10^−5^	0.99027	0.71287	0.29741
	CV	0.83065	0.031233	0.78558	0.35856
M	m.s.e.	0.24519	0.39634	0.67866	−0.29176
	CV	0.91041	0.011703	0.85654	0.067362
N	m.s.e.	0.74869	0.058151	0.94132	−0.086887
	CV	0.92505	0.0089125	1.2525	−0.53741
O	m.s.e.	0.2192	0.42644	0.44726	0.65783
	CV	0.25658	0.38381	−1.2194	−1.2194
P	m.s.e.	0.11362	0.57909	−0.74077	2.5079
	CV	0.76303	0.052963	0.94636	0.94636

**Table 3.** Regression statistics reveal a linear increase in m.s.e. with Froude number in only four subjects (subjects B, E, M, N). When assessed by CV, regression statistics reveal a linear increase in CV with speed in 10 subjects (subjects B, D, E, F, H, I, K, L, M, N, P).

subject	calculation	*R*^2^	*p*-values	slope	intercept
A	m.s.e.	0.0046152	0.91357	−1.5868	2.5799
	CV	0.12892	0.12892	1.2934	0.99583
B	m.s.e.	0.81487	0.035897	4.0503	1.6787
	CV	0.95911	0.0035539	4.8906	0.50204
C	m.s.e.	0.38129	0.26709	9.7009	2.2857
	CV	0.25265	0.38809	5.0405	0.76138
D	m.s.e.	0.15901	0.50608	10.397	2.0391
	CV	0.68014	0.085747	3.4016	3.4016
E	m.s.e.	0.59074	0.59074	2.7121	1.6061
	CV	0.72149	0.068545	2.6959	2.6959
F	m.s.e.	0.052231	0.71157	5.4128	5.4128
	CV	0.95626	0.23701	0.40558	0.96763
G	m.s.e.	0.12111	0.12111	0.56601	9.7704
	CV	0.29958	0.29958	1.3112	1.1867
H	m.s.e.	0.065915	0.065915	12.268	1.8277
	CV	0.70406	0.075589	3.0983	0.73232
I	m.s.e.	0.092519	0.61878	0.61878	3.759
	CV	0.79426	0.042363	2.4586	0.67764
J	m.s.e.	0.26784	0.26784	−6.0878	3.9844
	CV	0.23848	0.4039	4.7316	0.5203
K	m.s.e.	0.36064	0.36064	0.28421	−5.0511
	CV	0.75201	0.056934	2.3163	2.3163
L	m.s.e.	0.00038722	0.97495	2.0099	0.85545
	CV	0.78217	0.046352	2.7243	0.92868
M	m.s.e.	0.92868	0.43205	2.7945	0.12158
	CV	0.9031	0.9031	3.3641	0.68899
N	m.s.e.	0.78912	0.78912	2.8745	0.6905
	CV	0.6905	0.004444	4.5878	0.37161
O	m.s.e.	0.17444	0.48412	1.7153	0.98243
	CV	0.29152	0.34758	−4.6765	2.8734
P	m.s.e.	0.1053	0.5942	−2.5154	1.9703
	CV	0.74493	0.059546	3.2136	0.77527

## 4. Discussion

Variability is a fundamental feature of all biological systems [42,43] and, to our knowledge; this is the first attempt to understand the nature of variability in plantar pressure, and to quantify its relationship with walking speed. Our overall findings are that: both within and between subjects: (i) the absolute magnitude of variability in plantar pressure step-to-step is widely disparate across speeds, when calculated by m.s.e. (figures 1, 3 and 4), but low in absolute terms (less than 2.5%) when calculated by CV (figures 1, 3 and 5), (ii) RMA regression showed a linear increase in variability in plantar pressure by m.s.e. with speed and Froude number in only 4 subjects (*R*^2^ > 0.5, *p* = <0.05) (table 2) (figure 1), but in 11 subjects when assessed by CV with speed and Froude number (*R*^2^ > 0.5, *p* = <0.05) (table 3) (figure 3). Topological pixel-by-pixel analysis by pSPM did not provide any statistical support for an increasing or decreasing linear relationship between m.s.e. and walking speed (figure 2); and finally (iii) two commonly used metrics for quantifying variability provide directly contrasting pictures of how variability in plantar pressure step-to-step is spatially distributed across the plantar surface of the foot (figures 4 and 5). The main implication for these results is that when assessed by m.s.e., the relationship between variability in plantar pressure and speed, does not follow the commonly reported [1,4,12,34,41,44,45] biomechanical paradigm of variability measured in other lower limb kinematic parameters, that is, becoming more consistent at speeds faster or slower than comfortable walking speed.

### 4.1. Magnitude of variability

The inter-subject range of variability in m.s.e. is high. For example, the subject with the highest m.s.e. was 9.8× more variable in this parameter than the subject with the lowest m.s.e. at 1.5 m s^−1^ (figure 1). The subject with the highest CV was 2.3× more variable than the subject with the lowest CV at 1.5 m s^−1^ (figure 1). The m.s.e. is not used as a common calculator of variability in gait studies, and thus we cannot directly compare magnitudes derived here to values measured for other gait parameters. However, the large inter-subject variation in m.s.e. and CV is striking given our relatively homogeneous cohort of healthy adults, who ranged from 21 to 44 years old with no pathology or pre-existing injuries and a low BMI. CV less than 5% in kinematic step parameters is considered low [1,12,33,41,46–49], and less than 3% [4] very low. Here, we found variability in the kinetic step-parameter of plantar pressure assessed by CV was less than 2.5% across all subjects.

Low variability in plantar pressure can be understood by Bernstein's [50] dynamic systems theory. Coordination is defined by mastering redundant degrees of freedom to produce a controllable movement outcome [50]. The anatomical complexity of the foot provides multiple degrees of freedom, and optimal performance is achieved by exploiting available high levels of redundancy [11]. Variability in plantar pressure is low in order to coordinate the multiple available degrees of freedom in the foot complex, accounting for potential changes in substrate compliance, direction, speed and slope: all inevitable features of locomotion [33]. Controlled speed on a treadmill and thus, full sensorimotor awareness that there are no expected or unexpected changes in speed, substrate or direction, increases the pattern coordination of redundant degrees of freedom during normal walking, maintaining low variability. Each step cycle is thus assembled temporarily, but flexibly to facilitate adaptability, and maintain balance and stability [51]. It has been shown that when the sensorimotor system adopts functionally preferred states of coordination between soft and hard tissues it is ordered and stable, reflecting consistency in motor patterns [52] and thus low variability.

These results do not tend to support Todorov & Jordan's [11] theory that variability is higher in kinetic than kinematic parameters [11]. In their study of motor pattern behaviour using a computer controller, variability was higher in kinetic (specifically force and control signal variability) than kinematic features (joint angles), during a variety of hitting, trajectory and manipulative tasks. They suggested that higher variability in kinetics than kinematics is an underlying natural property of the mechanical system being controlled, rather than variability in the controller facilitating the movement [11]. However, in our study, variability in plantar pressure (kinetics) was consistently lower (less than 2.5%) than values considered very low (less than 3%) for variability of lower extremity kinematics. This difference might be attributed to the fact that our data reflect functional specifics of gait, whereas Todorov & Jordan's [11] data were derived from fine motor movements measured from a controller. Further investigation of this dataset is required to understand this specific relationship.

### 4.2. Variability–speed relationship

We found that variability in plantar pressure does not follow the same U-shaped curve relationship with speed, as do other lower limb step kinematic parameters. The strength of long-range correlations for each gait pattern consistently follows U-shaped curves, centred on the subjects preferred speed [5,53]. RMA regression statistics support an increasing variability in plantar pressure with speed when assessed by m.s.e. in only 4 out of 16 subjects, but in 10 out of 16 subjects when assessed by CV (*r*^2^ > 0.75, *p* ≤ 0.05] (table 2) (figure 1). When speed was normalized by Froude number, we found support for a linear increase in plantar pressure variability when assessed by m.s.e. in 4 subjects, and in 11 out of 16 subjects when assessed by CV (*r*^2^ > 0.75, *p* ≤ 0.05) (table 3) (figure 3). Almost all the same subjects had strong statistical support between comparisons (tables 2 and 3); however, subjects exhibiting strong statistical support were not consistently the same by speed or Froude number. All four subjects who exemplify strong supporting statistics showed an increasing linear relationship between variability in plantar pressure and speed when assessed by m.s.e. (tables 2 and 3) (figures 1 and 3).

The general lack of linear relationship was confirmed by topological pixel-by-pixel regression analysis of m.s.e. versus speed using pSPM, that revealed no statistical support for a linear increase or decrease in m.s.e. with speed (figure 2). Only pixels at the outer margin of the foot showed strong statistical linear trends, as a direct result of small changes in foot area contact size and shape that occurs step-to-step and at different speeds. Specifically, the red margins of the left inference prints in figure 2 indicate an increase in m.s.e. with speed around the periphery of the heel and forefoot, while the blue margins indicate a decrease in m.s.e. with speed around the periphery of the midfoot. It is likely that this significant relationship reflects changes in pressure or contact area increasing with speed in the forefoot and heel, and decreasing in pressure or contact area in the midfoot as speed increases. These artefacts could be removed by use of a non-rigid body image registration approach.

As discussed, walking speed has previously been shown to influence variability in lower limb step kinematics. Near the walk–run–walk transitions, stride duration increases before and after the transition [54], and increases at slower walking speeds (0.2–0.6 m s^−1^) compared with speeds of 0.8–1.4 m s^−1^. A linear increase in variability is present in joint angles [55], step length [41], step time interval [47] and step impulse [1]. Irrespective of the variable being measured, the data most commonly follow a U-shaped function at speeds faster and slower than comfortable walking speed, [1,53,54,56–58]. When younger and older adults are compared, stride time variability, and variability in frontal hip and knee motions, knee internal and external rotation, trunk motions [12] and step width [47] increased with speed in older adults. While the magnitude of variability is greater in older adults (more than 65), the variability–speed relationship is consistent between younger and older adults [12]. The observed difference here could be due to the fact that plantar pressure is not kinematic but kinetic, and this warrants further investigation.

### 4.3. Spatial distribution of variability

Finally, we presented in this contribution a novel means of analysis of quantifying the spatial distribution of step-to-step variation across the plantar surface using both m.s.e. and CV metrics. Using pSPM, we visualized variability in plantar pressure distribution in variation maps that plotted the mean m.s.e. and CV of each pixel from within the sample across the whole plantar surface of the foot, and combined the means to represent one print for each speed (figures 4 and 5). The m.s.e. variation maps show centres of highest variation to be highly localized under the lateral and medial forefoot, under metatarsal heads five and one (figure 4). However, equally, by the same qualitative assessment, CV variation maps show that the centres of highest variation lay more generally under the midfoot and phalanges (figure 5). This disparity may be largely explained by the differences between the two metrics and the ‘typical’ distribution of peak pressure across anatomical regions of the foot. The m.s.e. directly reflects the tendency for absolute peak pressure values to vary about the sample's absolute mean value, and may therefore be slightly susceptible towards bias indicating higher variation in areas of high absolute pressure. By contrast, CV represents a normalized measure of variability and shows a strong preference towards highlighting areas of low mean pressure as being highly variable. This generally explains why high CV values (red) are evident around the periphery of the foot and within the midfoot, and lower CV values (blue) are clustered where areas of high pressure exist, namely at the heel and forefoot (figure 5).

Although m.s.e. and CV appear qualitatively to paint opposing pictures of spatial variation, it is possible that the step-to-step variations they highlight are not biomechanically contradictory. A recent study found that pressure in the lateral forefoot was higher in steps where midfoot pressure was also elevated; and conversely that the same subjects exhibited statistically significant increases in pressure in the medial forefoot and hallux in steps where midfoot pressure was low [18]. Consistent with the latter finding, Stolwijk and co-workers [59] found that Malawian subjects with anatomically and/or functionally flatter feet also exhibited a more laterally placed centre of pressure in late stance. It is therefore possible that the variability in the midfoot highlighted by CV maps (figure 5) is functionally correlated to the forefoot variation seen in m.s.e. variation maps (figure 4), in terms of varying paths of the centre-of-pressure in mid- to late stance. Such a relationship would imply that the function of the midfoot and metatarsals one and five are highly interdependent.

Until we can obtain large samples of coronal transverse ground reaction force curves, we cannot draw firm conclusions. We suggest, however, that theoretically such a scheme of variability in mid- and forefoot pressure might reflect a biomechanical function for controlling perturbations in balance in late stance in the coronal plane, through internal/external foot rotation. It is further possible that variability in mid- and forefoot pressure relates to the mechanical influence of a tunable gearing ratio within the foot [60]: that is, the changing relative lengths of the muscle lever arm, and the load lever arm measured from the centre of pressure. Pre-tensioning, the effect of dorsiflexion at the talocrural joint on the plantar aponeurosis (PA) prior to heel strike [61], followed by a stretching of the PA around the metatarsal heads during toe-off (the windlass mechanism) [62], contributes to increased stiffening of the plantar soft tissues, and thus, increases the gear ratio at late stance [60]: when the centre of mass is over the forefoot. Hypothetically, it may be possible that the combined variation in gearing [60] and local stiffness [63]—with a potentially substantial contribution of varying tension of the transverse and oblique heads of abductor hallucis [64,65]—could contribute to the observed step-to-step variation in forefoot peak pressure, and to the extent to which medio-lateral transfer of pressure in late stance is achieved [65] (figure 4).

Using topological analysis, the spatial distribution of variability across the foot remains constant across walking speeds: areas of highest variability appear consistently confined to the lateral and medial forefoot (as calculated by m.s.e.; figure 4 and under the forefoot and heel (as calculated by CV; figure 5). This is perhaps surprising given that systematic changes in peak pressure distribution with speed have been consistently noted in the literature: that is, that peak plantar pressure is found to positively correlate with absolute and normalized walking speed in the heel and forefoot [25–30,66]. In addition to an overall increase in peak pressure, some have also noted decreasing pressure in the lateral midfoot, as a function of walking speed [18,22,66–68]; some suggesting a greater medial shift in centre of pressure as walking speed increases [64,65].

### 4.4. Future directions

This study offers the first quantitative and qualitative description of not only the observed variability in peak plantar pressure, but also the effect that speed has on the magnitude and spatial distribution of variability at the body–environment interface. Future work should consider comparable and quantifiable ways to compare kinetic and kinematic parameters, and the effects of sample size and step-to-step variation on statistical comparisons of foot pressure. Analysis of variability in plantar pressure in older people and people at risk of foot pathology such as diabetics, could assist in determining the impact of neuromuscular and sensorimotor decline on variability in plantar pressure distribution and its relationship to speed across ontogeny. Systematic changes in peak pressure across ontogeny have been demonstrated in a number of studies, and further insights into foot function may be gleaned if relatable changes in variability were found to exist. Tunable gearing in children has been shown to mature quite late: consistently low forefoot plantar pressures compared with adults [20] are recorded in children under 5 years old, and there is late development of the medial-to-lateral transfer of the centre of pressure [69]. Functionally, the forefoot delivers propulsive force from the hind- to the forefoot, facilitating toe-off; however, before 7–8 years of age, accelerative power is driven from the hind- and midfoot [70]. That we found variability in peak pressure is highest in the lateral and medial forefoot (assessed by m.s.e.), and more generally in the forefoot (assessed by CV), is consistent with the findings of Li and co-workers [70]. Finally, a similar analysis should be completed during non-treadmill walking at comfortable walking speeds, and on uneven terrain, to ascertain whether variability increases or decreases when speed is not controlled, thus testing dynamic system theory.

## 5. Conclusion

Two measures of variability were used in this paper: one, a standard mathematical formula to assess variability (m.s.e.), and another, a measure commonly applied to clinical questions (CV). We conducted experiments solely on healthy young subjects, observing disparate levels of variability consistently confined to the medial and lateral forefoot as assessed by m.s.e., and low levels of variability (less than 2.5%) in the forefoot and heel as assessed by CV. From these results, we determine that the magnitude of variability assessed by CV is generally dependent on speed, while the spatial distribution of variability in plantar pressure when assessed by m.s.e. is independent of speed. This paper presents the first attempt to understand not only the nature of variability in plantar pressure, but also the influence that speed has over the magnitude and spatial distribution of variability in plantar pressure. Functional variability and the exploitation of functional redundancy are products of an adaptive biomechanical system driven by motor control. Given the tendency for degradation of biomechanical systems, the study of variability and its contribution to balance and stability in gait across ontogeny, is an essential feature when understanding how to prevent, resist and recover from instability events during gait.

## Data accessibility

Data can be accessed at doi:10.5061/dryad.09q2b.
